# Supplementary material for: EnzML: multi-label prediction of enzyme classes using InterPro signatures
Source: BMC Bioinformatics. 2012 Apr 25;13:61. doi: 10.1186/1471-2105-13-61 (PMC3483700; doi:10.1186/1471-2105-13-61)
Supplement: Addtional file 5 — The Java code to format the data files, evaluate and predict. The file enzml_java_code.tar.gz contains the Java code used to format database data to ARFF and XML formats, to execute cross and train-test (jackknife) evaluations and to record evaluation results to database. More information is included in the readme.txt file and the Javadoc files. The code can be used with a MySQL database. To use a different database software, other JDBC drivers might be required. [file 1471-2105-13-61-S5.gz › java_code/utils/doc/uk/ac/ed/inf/utils/database/TableColumn.html]

TableColumn


---


|  |  |  |
| --- | --- | --- |
| **Overview** \| **Package** \| **Class** \| **Use** \| **Tree** \| **Deprecated** \| **Index** \| **Help** | |  |
| **PREV CLASS**   **NEXT CLASS** | **FRAMES**    **NO FRAMES**     **All Classes** |  |
| SUMMARY: NESTED \| FIELD \| CONSTR \| METHOD | DETAIL: FIELD \| CONSTR \| METHOD |  |


---


## uk.ac.ed.inf.utils.database Class TableColumn

```
java.lang.Object
  uk.ac.ed.inf.utils.database.TableColumn
```

**All Implemented Interfaces:**: java.lang.Comparable<TableColumn>

---

``` public class TableColumn extends java.lang.Object implements java.lang.Comparable<TableColumn> ```

A column object contains all the information to create, fill and delete a
database table column

**Version:**
:   24-Mar-2007

**Author:**
:   Luna - luna.deferrari@ed.ac.uk

---

**Constructor Summary**

`TableColumn(java.lang.String columnName,
java.lang.String dataType,
boolean isKey)`
  
          Constructor 2: takes the column name, data type and primary key status


| **Method Summary** | |
| --- | --- |
| `int` | `compare(java.lang.Object f1, java.lang.Object f2)`             Compares two columns by their name, returns -1 if f1 comes alphabetically before f2, 0 if the two are equal and +1 if f2 comes alphabetically before f1. |
| `int` | `compareTo(TableColumn f2)`             Compares two columns by their name, returns -1 if this column comes alphabetically before f2, 0 if the two are equal and +1 if f2 comes alphabetically before this column. |
| `boolean` | `equals(TableColumn anotherColumn)`             Compares two columns by their name, returns true if the two columns have the same name, false otherwise. |
| `java.lang.String` | `getColumnName()`             Gets the column name |
| `java.lang.String` | `getDataType()`             Gets the column data type |
| `int` | `getNoOfRows()`             Gets the number of rows in the column |
| `boolean` | `isPrimaryKey()`             Gets the column primary key status |
| `void` | `setIsPrimaryKey(boolean isKey)`             Sets the column primary key status |

**Methods inherited from class java.lang.Object**

`equals, getClass, hashCode, notify, notifyAll, toString, wait, wait, wait`

**Constructor Detail**

### TableColumn

```
public TableColumn(java.lang.String columnName,
                   java.lang.String dataType,
                   boolean isKey)
```

:   Constructor 2: takes the column name, data type and primary key status

    **Parameters:**: `columnName` - name of the column: `dataType` - data type (e.g. varchar(25) ): `isKey` - true if the column is a primary key


**Method Detail**

### compare

```
public int compare(java.lang.Object f1,
                   java.lang.Object f2)
```

:   Compares two columns by their name, returns -1 if f1 comes alphabetically
    before f2, 0 if the two are equal and +1 if f2 comes alphabetically
    before f1.

    :   **Parameters:**: `f1` - the first column to compare: `f2` - the second column to compare **Returns:**: returns -1 if this column is smaller than f2, 0 if equal and +1 if greater (in alphabetical order)

---


### compareTo

```
public int compareTo(TableColumn f2)
```

:   Compares two columns by their name, returns -1 if this column comes
    alphabetically before f2, 0 if the two are equal and +1 if f2 comes
    alphabetically before this column.

    :   **Specified by:**: `compareTo` in interface `java.lang.Comparable<TableColumn>`
    :   **Parameters:**: `f2` - the second column to compare **Returns:**: returns -1 if this column is smaller than f2, 0 if equal and +1 if greater (in alphabetical order)

---


### equals

```
public boolean equals(TableColumn anotherColumn)
```

:   Compares two columns by their name, returns true if the two columns have
    the same name, false otherwise.

    :   **Parameters:**: `anotherColumn` - the second column to compare **Returns:**: returns true if the two columns have the same name, false otherwise.

---


### getColumnName

```
public java.lang.String getColumnName()
```

:   Gets the column name

    :   **Returns:**: the column name

---


### getDataType

```
public java.lang.String getDataType()
```

:   Gets the column data type

    :   **Returns:**: the column data type, e.g. VARCHAR(10)

---


### getNoOfRows

```
public int getNoOfRows()
```

:   Gets the number of rows in the column

    :   **Returns:**: the number of rows in the column

---


### isPrimaryKey

```
public boolean isPrimaryKey()
```

:   Gets the column primary key status

    :   **Returns:**: the primary key status, e.g. 'true'= is a primary key

---


### setIsPrimaryKey

```
public void setIsPrimaryKey(boolean isKey)
```

:   Sets the column primary key status

    :   **Parameters:**: `isKey` - the primary key status, e.g. 'true'= is a primary key


---


|  |  |  |
| --- | --- | --- |
| **Overview** \| **Package** \| **Class** \| **Use** \| **Tree** \| **Deprecated** \| **Index** \| **Help** | |  |
| **PREV CLASS**   **NEXT CLASS** | **FRAMES**    **NO FRAMES**     **All Classes** |  |
| SUMMARY: NESTED \| FIELD \| CONSTR \| METHOD | DETAIL: FIELD \| CONSTR \| METHOD |  |


---
